# 3D bioprinting in cardiac tissue engineering

**DOI:** 10.7150/thno.61621

**Published:** 2021-07-06

**Authors:** Zihan Wang, Ling Wang, Ting Li, Sitian Liu, Baolin Guo, Wenhua Huang, Yaobin Wu

**Affiliations:** 1 Guangdong Engineering Research Center for Translation of Medical 3D Printing Application, Guangdong Provincial Key Laboratory of Medical Biomechanics, Department of Human Anatomy, School of Basic Medical Sciences, Southern Medical University, Guangzhou, 510515, China.; 2 Guangdong Medical Innovation Platform for Translation of 3D Printing Application, Southern Medical University, The third Affiliated Hospital of Southern Medical University, Southern Medical University, Guangzhou, 510515, China.; 3 Biomaterials Research Center, School of Biomedical Engineering, Southern Medical University, Guangzhou, 510515, China.; 4 The First School of Clinical Medicine, Southern Medical University, Guangzhou, 510515, China.; 5 Frontier Institute of Science and Technology, and State Key Laboratory for Mechanical Behavior of Materials, Xi'an Jiaotong University, Xi'an, 710049, China.

**Keywords:** 3D bioprinting, bioinks, printed biomaterials, cardiac muscle, tissue engineering

## Abstract

Heart disease is the main cause of death worldwide. Because death of the myocardium is irreversible, it remains a significant clinical challenge to rescue myocardial deficiency. Cardiac tissue engineering (CTE) is a promising strategy for repairing heart defects and offers platforms for studying cardiac tissue. Numerous achievements have been made in CTE in the past decades based on various advanced engineering approaches. 3D bioprinting has attracted much attention due to its ability to integrate multiple cells within printed scaffolds with complex 3D structures, and many advancements in bioprinted CTE have been reported recently. Herein, we review the recent progress in 3D bioprinting for CTE. After a brief overview of CTE with conventional methods, the current 3D printing strategies are discussed. Bioink formulations based on various biomaterials are introduced, and strategies utilizing composite bioinks are further discussed. Moreover, several applications including heart patches, tissue-engineered cardiac muscle, and other bionic structures created *via* 3D bioprinting are summarized. Finally, we discuss several crucial challenges and present our perspective on 3D bioprinting techniques in the field of CTE.

## Introduction

**Figure 1:**
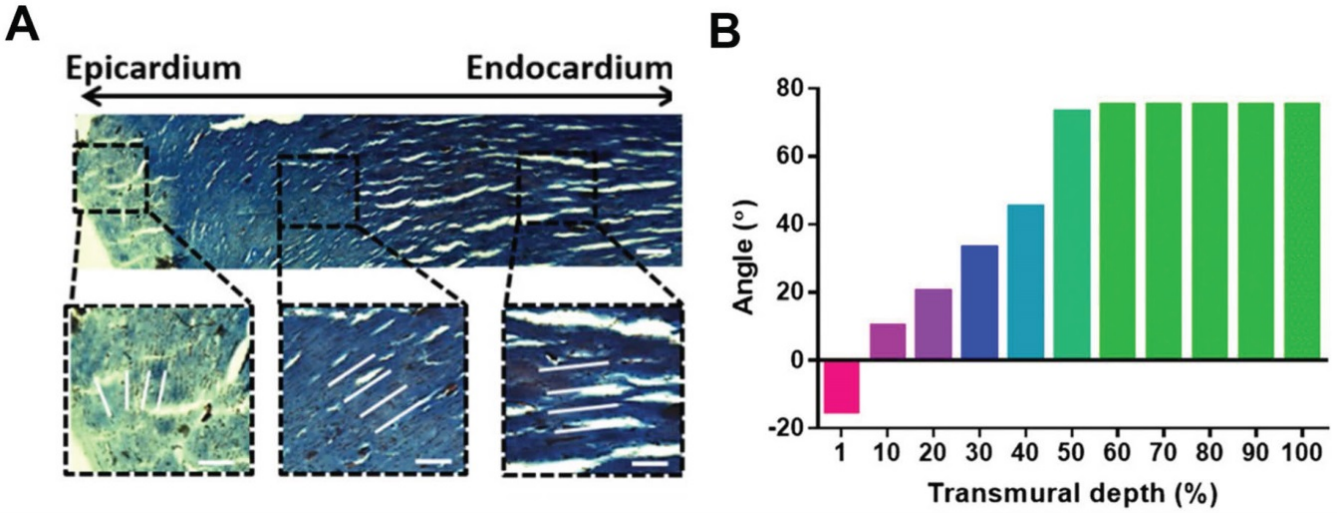
Adult rat hearts were harvested, sliced, and stained for cells and collagen to analyze variations in the transmural orientation. (A) Masson's trichrome staining of a transmural block cut from the ventricular wall showing the macroscopic variation in fiber orientation across the wall. (B) Analysis of collagen fiber orientation revealed that the degree of alignment from the epicardial side to the endocardial side had a 100° shift. Adapted with permission from 20. Copyright 2017, National Academy of Sciences.

Cardiovascular diseases are highly prevalent diseases worldwide in terms of morbidity and mortality, especially in developed countries 1. One out of every seven deaths is caused by coronary artery disease, and the estimated yearly incidence of myocardial infarction (MI) is 790,000 1-3. The adult human heart is the least regenerative organ in the body due to the limited regeneration capacity of cardiomyocytes (CMs) 4, 5, which means the heart cannot repair itself after injury 6. Although various therapy strategies, such as heart stents and coronary bypass surgery, have been implemented in the clinic, reversing myocardial deficiencies remains an ongoing challenge. Cardiac tissue engineering (CTE) aims to develop functional engineered tissues and organs as *in vivo* transplants to alleviate the shortage in organs for transplantation or as *in vitro* models for research on disease mechanisms and drug discovery 7-9. The native heart is a muscular organ that pumps blood through the blood vessels of the circulatory system 10. The wall of the heart is composed of three layers: epicardium, myocardium, and endocardium (from the outside to the inside). The myocardium contains multiple anisotropic layers of CMs and collagen fibers with a gradual transition in orientations across its transmural depth, which contributes to the unique biomechanical behavior of cardiac tissue (**Figure 1**) 11. The myocardium contains 2-4 billion aligned CMs (roughly 75% of the heart volume) although they represent only about 33% of the total cell number 12. In addition, CMs associate with other types of cells, including endothelial cells (ECs), smooth muscle cells, and fibroblasts (FBs), to generate an intricately organized 3D structure 13. Cardiac muscle also contains specialized cells that rapidly conduct electrical signals called Purkinje cells. Coronary arteries and countless capillaries are also present to nourish thicker cardiac tissues 14. Thus, native cardiac muscle tissue is a relatively complicated system containing many features. Therefore, engineered cardiac tissue requires, at minimum, active cardiac cells with precise cell alignment and a 3D extracellular environment with specific cardiac structure and involvement of non-myocyte cells.

In the past decade, many studies have proved that prepared 3D culture environments are closer to *in vivo* conditions than conventional 2D culture techniques (Petri dishes) 15. Many bioengineering techniques have been developed to generate elaborate and functional engineered cardiac tissue including micropatterning 16, electrospinning 17, and 3D bioprinting (3DBP). 3DBP is regarded as an especially promising technique for fabricating biomimetic scaffolds with the complex 3D structures required for cell proliferation and differentiation in various tissue engineering applications. Previous studies have reviewed 3DBP for cardiovascular regeneration and CTE applications including the development of printing parameters and bioink materials 18, 19. In this review, we first briefly summarize published studies in CTE based on traditional scaffold fabrication techniques, and then demonstrated 3DBP would be beneficial for overcoming the drawbacks of the traditional CTE scaffolds. Subsequently, we describe various 3DBP strategies, biomaterials-based bioinks, and cell sources for 3DBP CTE. We also highlight the current application of 3DBP in CTE. Finally, we discuss the major challenges of current 3DBP strategies, describe future trends, and provide our perspective on the potential of bioprinted cardiac constructs for advancing research and clinical applications.

## Overview of cardiac tissue engineering (CTE)

Tissue engineering scaffolds play a key role in CTE applications because they offer a supportive framework and provide a promotive micro-environment for cell adhesion, proliferation, and differentiation. The following scaffold properties are important for CTE applications: (i) Biocompatibility: scaffolds should show low immunogenicity and coagulability when implanted *in vivo*. (ⅱ) Biodegradability: the transplanted scaffolds should be degraded *in vivo* by hydrolysis, oxidation, enzymes, or physical degradation. (ⅲ) Mechanical strength: scaffolds should retain the shape with muscle tissue-like stiffness (~10 kPa) 21. (ⅳ) Bioactivity: scaffolds should enhance cell adhesion with high activity and promote cell proliferation and differentiation. Excellent scaffolds mimic the cellular components and specific micro-environment of the native tissue, such as its cell arrangement, micro-extracellular matrix (ECM) composition, and physical properties 22-24. (ⅴ) Electrical conductance: scaffolds should allow the engineered constructs to perform the dynamic functions of the heart 25. (vi) Anisotropy: scaffolds should have an anisotropic microstructure, which has been shown to promote CM alignment and favor cell differentiation and functionality 26-30.

**Figure 2:**
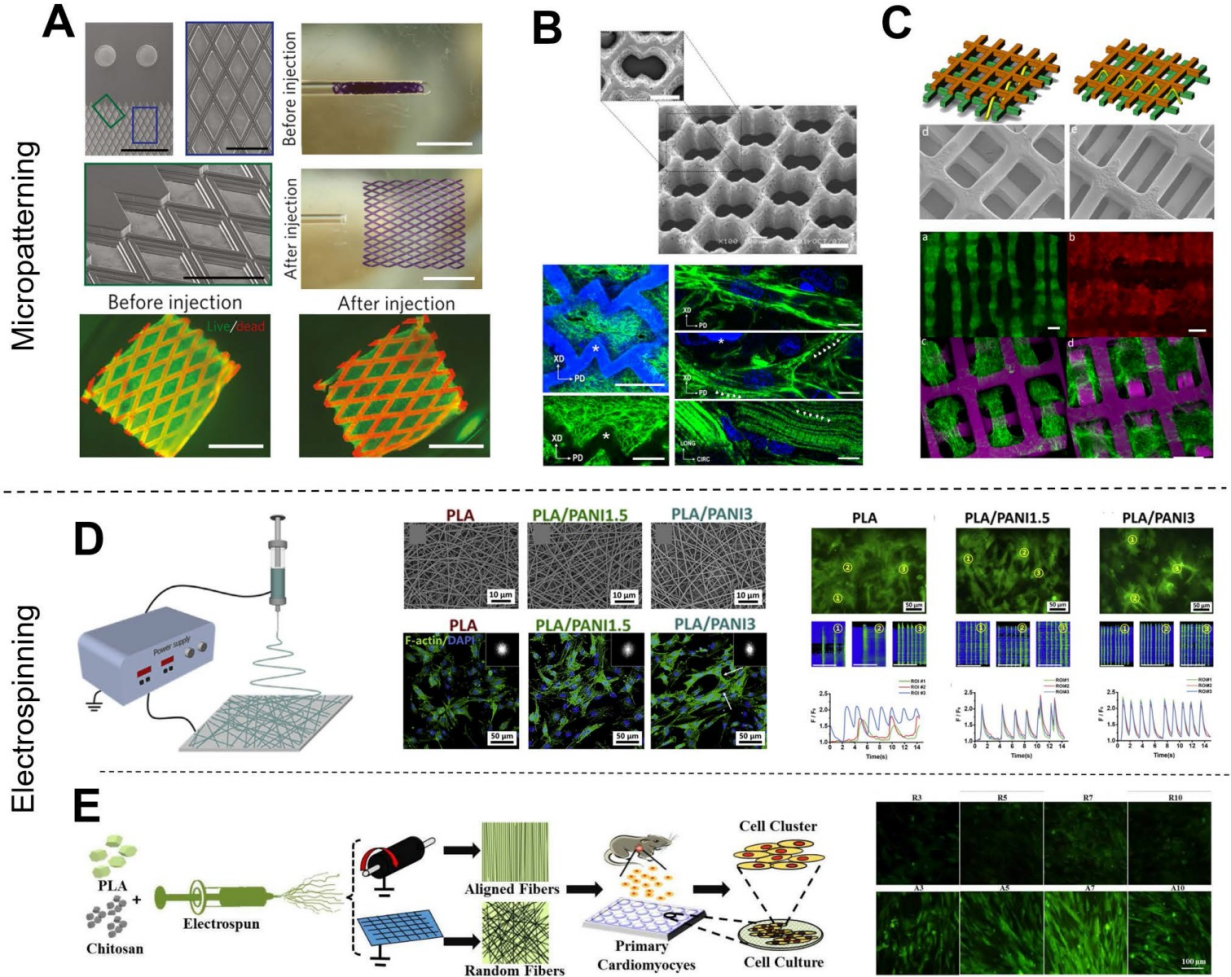
Traditional fabrication methods in CTE. (A-C) Examples of micropatterned scaffolds fabricated from bioelastomers. (A) Grid-patterned scaffold for myocardial repair prepared on a polydimethylsiloxane (PDMS) mold. Adapted with permission from 37. Copyright 2017, Springer Nature. (B) Accordion-like honeycomb CTE scaffolds fabricated by excimer laser microablation. Adapted with permission from 38. Copyright 2008, Springer Nature. (C) Multi-layered micropatterned elastic CTE scaffold fabricated using a microelectromechanical technique and packaging approach. Adapted with permission from 39. Copyright 2013, John Wiley & Sons. (D-E) Examples of electrospun nanofibrous scaffolds for CTE applications. (D) Conductive electrospun nanofibrous sheet based on poly(L-lactic acid)/polyaniline. The scaffold promoted the maturation and spontaneous beating of primary CMs. Adapted with permission from 48. Copyright 2017, Elsevier. (E) Random or aligned electrospun nanofibrous mats based on PLLA/chitosan. These scaffolds showed promise as platforms for regenerating myocardia and drug screening applications. Adapted with permission from 35. Copyright 2017, Elsevier.

Various methods have been developed to fabricate scaffolds that induce cell orientations including micropatterning 16, 31-34, and electrospinning 17, 28, 35, 36. Micropatterning is a simple and effective technique to fabricate anisotropic patterns for cell alignment. Various scaffold micropatterns have been developed for CTE applications, such as grids (**Figure 2A**) 37, accordion-like honeycombs (**Figure 2B**) 38, and even multi-layered patterns (**Figure 2C**) 39. In particular, our previous study showed that micropatterned electroactive bioelastomer films (groove/ridge = 50 μm/50 μm) were able to significantly guide the elongation and alignment of primary CMs, and increased the intercellular concentration of Ca^2+^ compared with flat films 16. Other studies 40, 41 have applied honeycomb microstructures as CTE scaffolds because cardiac muscle fibers are surrounded and coupled by endomysial collagen sheaths that are bundled within a honeycomb-like network of undulated perimysial collagen fibers 42. Compared to square and rectangular patterns, honeycomb patterns generated scaffolds with higher ultimate tensile strength and strain to failure 41, 43. Additionally, electrospinning is an excellent technique for fabricating nanofibrous scaffolds for CTE 44. Electrospinning generates scaffolds with excellent mechanical properties and provides easy manipulation of fiber properties, great material handling, and scalable production. Studies have reported that fibers with decreased diameter facilitate cell adhesion and spreading 45 because cell membranes with embedded receptors can easily wrap around sub-micron-scale fibers 46, 47. We previously reported that electrospun conductive nanofibrous sheets were able to enhance cell-cell interactions, maturation, and spontaneous beating of primary CMs (**Figure 2D**) 48. Also, available electrospinning collectors enable the production of a variety of nanofibrous constructs 36. For example, flat collectors are used to generate random fibers while high-speed rotating drum and mandrel collectors are used to generate aligned fibers (**Figure 2E**) 48-50. In our other previous studies, we used a developed dry-wet electrospinning method to develop a list of aligned nanofiber yarn (NFY) 34, 51. In summary, these published studies based on micropatterning and electrospinning techniques demonstrated induction of cell alignment on 2D substrates or pseudo-3D environments. However, recapitulating the 3D organized cellular architecture of native heart tissues using these techniques remains challenging 6.

**Figure 3:**
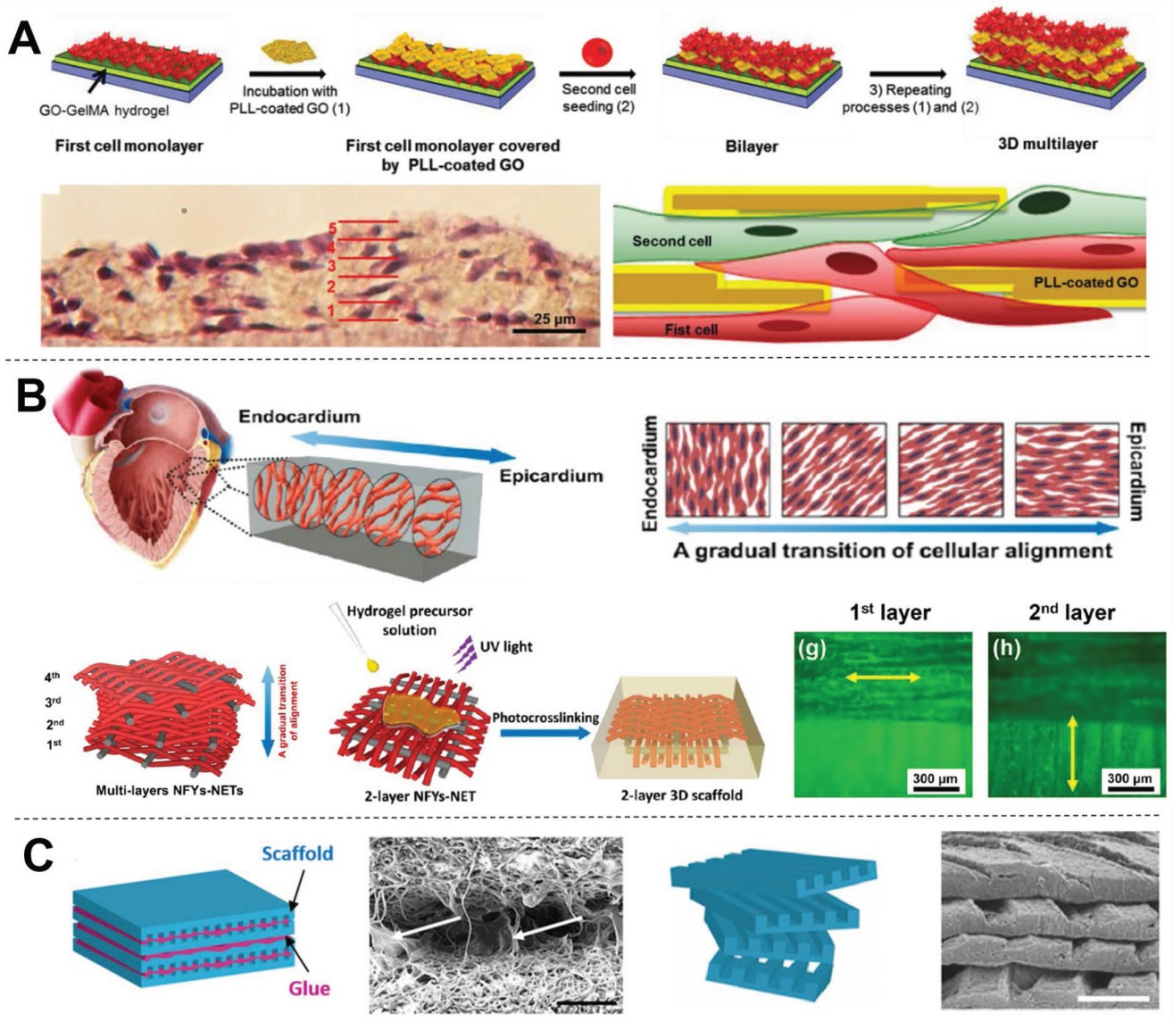
Examples of 3D CTE scaffolds with multi-layered structures (A) Multi-layer 3D CTE scaffold fabricated *via* layer-by-layered deposition of cardiac cells and graphene oxide (GO)-coated poly-L-lysine (PLL) films. Adapted with permission from 59. Copyright 2014, John Wiley & Sons. (B) Multi-layered NFYs/hydrogel core-shell scaffold within a transition in the 3D orientation of CMs. Adapted with permission from 11. Copyright 2017, American Chemical Society. (C) Grooved electrospun scaffolds stacked with a slight angular shift for guiding the orientation of multi-layered CMs. Adapted with permission from 20. Copyright 2017, National Academy of Sciences.

Hydrogel matrix systems present a 3D environment similar to that of native tissues, and cells can be encapsulated within hydrogel matrices homogeneously. Moreover, external stimulation approaches such as mechanical stimulation or electrical stimulation have been applied to control 3D cell alignment and elongation within hydrogel scaffolds 52-55. However, the inconvenience of these external stimulation methods limits their application 56-58. Shin *et al.* 59 presented an interesting method for engineering 3D multi-layered constructs using layer-by-layer assembly of cells separated by self-assembled graphene oxide-based thin films (**Figure 3A**). This multi-layered construct showed strong spontaneous beating and frequency-dependent opening/closing actuation under a low external electric field. However, CMs within the structure presented a random orientation, which is detrimental to heart contraction. In contrast, we previously developed core-shell scaffolds with electrospun aligned nanofiber yarns (NFYs) as the core and hydrogel as the shell that not only induced cell alignment and elongation but also provided a suitable 3D environment for cellular nutrient exchange and mechanical protection 34. Furthermore, we designed an interwoven NFYs/hydrogel core-shell scaffold that controlled cell alignment and elongation according to the complex interwoven structure of native cardiac tissue, and then stacked cell-laden NFYs *via* layer-by-layer assembly with a slight angular shift to mimic the gradual transition in alignment between myocardium layers (**Figure 3B**) 11. Similarly, Fleischer *et al.* 20 fabricated albumin micro-grooved scaffolds *via* electrospinning and laser patterning to engineer aligned cardiac tissues, and then stacked several of these grooved scaffolds with a slight angular shift (**Figure 3C**). Unfortunately, this layer-by-layer stacking strategy significantly increases the complexity of cell culture, and precise 3D cell and matrix organization is limited. In contrast, 3D printing showed the advantages for fabricating scaffolds with 3D complex structure, and these drawbacks of the traditional scaffolds are now being overcome by computer-assisted 3DBP 60.

## 3D bioprinting techniques for engineered cardiac tissue

3DBP, which similar to traditional 3D printing, is also an additive manufacturing technique but the printed inks or resins are replaced with cells only or biomaterials and cells mixture 61, 62. 3DBP spatially controls the deposition of bioinks, allowing for the fabrication of functional living constructs with 3D customized architecture. Recent advancements have enabled several novel printing strategies.

### 3D bioprinting approaches

#### Inkjet printing

**Figure 4:**
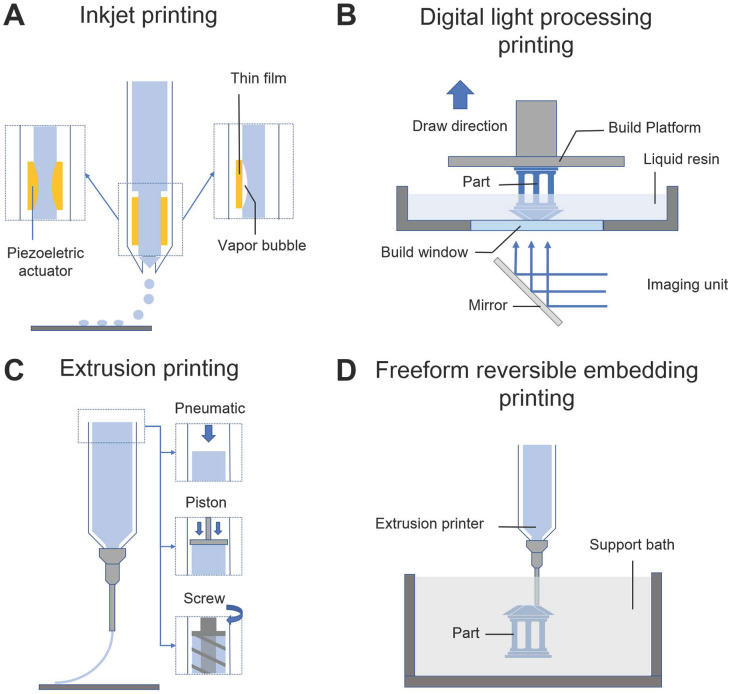
Schematic illustrations of various 3DBP technologies. (A) Inkjet printing: thermal or piezoelectric actuators are applied to form droplets of bioink-cell hybrids. (B) Digital light processing printing: ultraviolet or visible light is used to cure a photopolymer in a vat for layer-by-layer manufacturing of a 3D model. (C) Extrusion printing: pneumatic, piston, or screw forces are applied to extrude continuous beads of bioink. (D) Freeform reversible embedding printing: bioink is extruded into a reversible support bath for fabrication of support-free structures.

2D inkjet printers are modified for 3DBP by replacing the ink cartridge with biological material and adding an electronically controlled elevator stage for z-axis movement (**Figure 4A**). Inkjet bioprinting devices are classified into two types including thermal 63 and piezoelectric ones 64, 65. Thermal-based printers produce pressure pulses and eject droplets by localized heating, while piezoelectric-based printers generate acoustic waves. The heat and acoustic waves have a negligible impact on cell viability 66, 67. Inkjet bioprinting is compatible with many biomaterials and can maintain remarkable cell viability (> 90%) 68. Boland's group used modified inkjet printers to fabricate contractile cardiac hybrids in the form of 3D rectangular sheets and half hearts 69, 70. Moreover, the cardiac hybrid materials were tailored in 3D to achieve desired porosities and mechanical and chemical properties. However, considering its operating principle, inkjet-based bioprinting works with bioinks having a specific viscosity (3.5-12 mPa/s) 61, which usually leads to weak mechanical support 65. To overcome this physical limitation, a post-crosslinking strategy has been introduced 71, 72. Nevertheless, inkjet printers cannot still print cells at high densities, which is critical for creating cardiac constructs 73, 74.

#### Digital light processing printing

In digital light processing (DLP) printing, a digital micromirror array device is used to selectively solidify photocurable bioinks in a layer-by-layer process controlled by a moveable stage along the z-axis (**Figure 4B**) 75. The main advantage of DLP printing is its simple and rapid manufacturing process. As the entire layer is printed simultaneously, the motion of the printer head in the x-y direction is avoided. This nozzle-free approach also avoids clogging and excessive shear stress to cells. Furthermore, it provides a higher resolution (50-100 μm) than the other printing strategies 6. Liu *et al.* 76 encapsulated neonatal mouse ventricular CMs within a photocurable hydrogel by a DLP printing approach. The encapsulated CMs were aligned with the printed microarchitecture and showed observable force trace after various stimulation frequencies *in vivo*, which indicated that the rapid DLP printing approach was able to fabricate complex CTE scaffolds. However, several limitations still need to be overcome to apply DLP printing to CTE. First, biomaterials used for DLP printing must be photocurable; however, UV light exposure potentially damages cell DNA during the 3D printing process 77. In addition, cell settling or sedimentation during DLP bioprinting also should be discussed, particularly when a thick scaffold is needed for printing. Chan *et al.* 78 found that, in a typical DLP printing approach, cells mixed within the bioresin settled to the bottom of the bioresin reservoir during printing, which created an inhomogeneous cell distribution within the printed construct. To overcome this challenge, Lin *et al.* 79 matched the buoyant density of the cells by adding 37.5% (v/v) Percoll to a poly(ethylene glycol) (PEG)-based bioresin to prevent cell settling. Moreover, it remains challenging to utilize multiple types of bioinks in DLP printing 80. In comparison, extrusion printing can be used to easily fabricate constructs of multiple cells/materials by equipping two or more printer heads 81. However, a multiple bioresin reservoirs strategy is not suitable for DLP printing because of inevitable cross pollution between the reservoirs.

#### Extrusion printing

Extrusion printing uses pneumatic- or mechanical-driven fluid dispensing systems to constantly extrude bioink onto a platform (**Figure 4C**), which permits faster, simpler, and more affordable bioprinting compared with other techniques. Moreover, the deposited cell densities could be high and close to physiological CM densities (10^8^-10^9^ cells mL^-1^), which is particularly crucial for CTE 73, 74. However, the dispensing pressures and shear stresses applied to cells are significant and may result in poor cell survival (40-80 % cell viability) when extruding materials with high viscosity (> 6 × 10^7^ mPa s) or when a thin nozzle is used 82, 83. One study showed that cardiac myocytes are more sensitive to extrusion pressure than cardiac fibrocytes 84. Therefore, shear-thinning hydrogels have been developed to overcome this limitation. Unlike conventional Newtonian bioinks, shear-thinning bioinks show significantly decreased viscosity with increasing strain rate during printing, which protects the cells from high shear stresses. For example, gelatin and its derivate materials as well as some decellularized ECM (dECM) hydrogels 74 are shear-thinning bioinks that are widely used in 3DBP for tissue engineering.

#### Freeform reversible embedding printing

**Figure 5:**
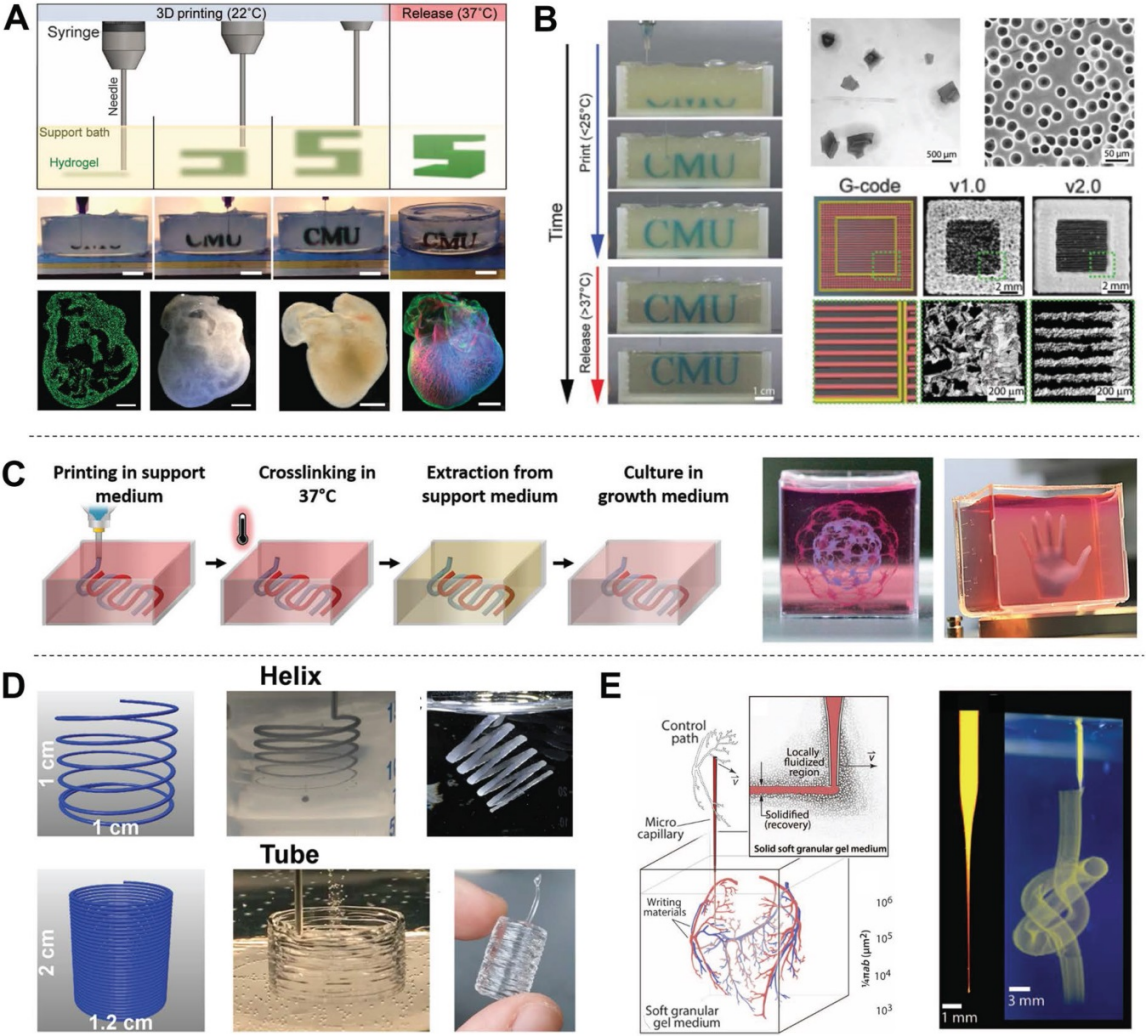
Examples of FRE printing strategies. (A) Schematic of the printing and crosslinking process of hydrogel (green) within a gelatin slurry support bath (FRESH v1.0). A whole neonatal-scale human heart was printed via FRESH v1.0. Adapted with permission from 86. Copyright 2015, American Association for the Advancement of Science. (B) Gelatin microspheres with smaller diameter were used as the support bath in FRESH v2.0, resulting in printed structures with higher resolution. Adapted with permission from 85. Copyright 2019, American Association for the Advancement of Science. (C) A xanthan gum support bath was shown to support complicated structures (hollow sphere, small hand). Adapted with permission from 91. Copyright 2019, John Wiley & Sons. (D) Helical and tubular structures printed in Carbopol support gel by freeform extrusion. Adapted with permission from 89. Copyright 2016, American Chemical Society. (E) A continuous knot of aqueous fluorescent microspheres in Carbopol support gel written without the simultaneous building of any other support structure. Adapted with permission from 90. Copyright 2015, American Association for the Advancement of Science.

Hydrogels are excellent candidates for supporting cell proliferation in 3D environments. However, most hydrogels suffer from poor mechanical properties, which potentially results in a collapse during printing of hollow structures due to gravity 85. Thus, direct printing of hydrogels and other soft biomaterials (e.g., dECM) remains challenging. To overcome this limitation, Feinberg *et al.* 86 developed a strategy to extrude hydrogel bioink into another hydrogel as a support medium, which they called freeform reversible embedding (FRE) of suspended hydrogels (FRESH) (**Figure 4D**). The support bath in this strategy was composed of gelatin microparticles and acted like a Bingham plastic during printing. Bingham plastics behave as a rigid body at low shear stresses but flow as a viscous fluid at high shear stresses. This meant that there was little mechanical resistance when a needle-like nozzle moved through the bath, yet hydrogel extruded out of the nozzle and deposited within the bath was held in place. Thus, printed soft materials maintained the intended 3D geometry in this support bath. After solidification, the printed scaffold was easily taken out by melting the support bath (**Figure 5A**). In a subsequent study, Feinberg's group decreased the size of the gelatin microparticles in the support bath from ~65 μm to 25 μm and renamed the system FRESH v2.0 (**Figure 5B**) 85. The diameters of the printed collagen filaments reliably decreased from 200 μm to 20 μm when FRESH v2.0 was used. Moreover, a porous microstructure was present after the gelatin microspheres were removed from the 3D-printed scaffold, which promoted cell infiltration and micro-vascularization 87. Recently, various hydrogel materials have been developed as support media based on this strategy. For example, Edri *et al.* 88 printed two homocentric hollow spheres and a small hand in xanthan gum as the support bath (**Figure 5C**). Hinton *et al.* 89 printed helical and tubular structures in a Carbopol support gel (**Figure 5D**). FRE printing also has great potential for the fabrication of complicated structures. For example, Bhattacharjee *et al.* 90 printed a continuous knot in Carbopol support gel (**Figure 5E**). Even a whole neonatal-scale human heart has been successfully printed 85. However, the conditions of the support bath (e.g., temperature, pH, ion concentration) might affect cell activity, especially prolonged printing. Another limitation is that the integrity of delicate structures and the viability of sensitive cells may be jeopardized by the mechanical force needed to remove the support medium 91. Thus, compatible support baths are still very limited in variety.

### Bioink preparation

Various biomaterials have been applied as bioinks for CTE applications recently** (Table 1)** 101, 102. Usually, bioinks are made of hydrogel or polymer and cells are encapsulated into the bioinks or seeded on bioprinted constructs to promote cardiac tissue growth. The chosen biomaterials should be printable, which indicates their suitability for the fabrication of stable 3D constructs with high structural integrity and fidelity. Therefore, the mechanical properties of bioinks (e.g., modulus, rheology) are fundamental. In general, bioinks should provide a constant, precise deposition, followed by rapid, nontoxic solidification. These are key factors affecting resolution, cell viability, and post-print function, although each printing strategy has a few differences in detail 102-104. The chosen biomaterials must also shield cardiac cells against varying levels of pressure and shear stress developed during printing processes 105. Biomaterials also need to mimic the ECM of the human heart tissue to promote cell proliferation and differentiation 74. Generally, biomaterials used as bioinks are grouped into natural and synthetic materials 106.

#### Natural biomaterials as bioinks

**Figure 6:**
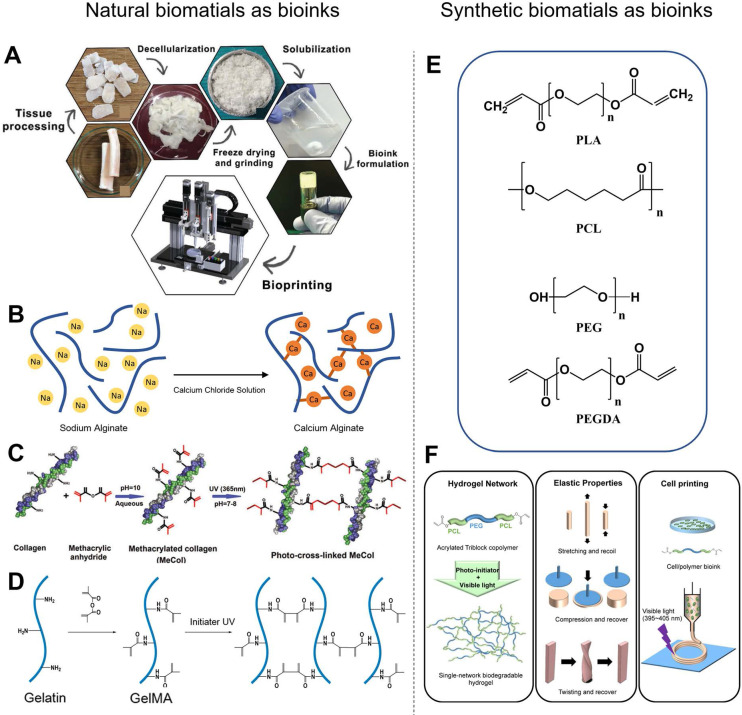
Examples of natural and synthetic biomaterials applied as bioinks. (A) Schematic diagram of the process for preparing dECM bioink. Adapted with permission from 116. Copyright 2018, John Wiley & Sons. (B) Schematic diagram of alginate calcium-induced crosslinking. (C) Schematic diagram of GelMA formation and UV light-induced crosslinking. Adapted with permission from 97. Copyright 2018, Mary Ann Liebert. (D) Schematic diagram of MeCol formation and UV light-induced cross-linking. (E) Chemical structures of synthesized polymers commonly used in 3DBP. PLA, polylactic acid; PCL, polycaprolactone; PEG, poly(ethylene glycol); PEGDA, poly(ethylene glycol) diacrylate. (F) Schematic diagram of acrylated PCL-PEG-PCL triblock polymer synthesis, and simple gelation into single-network hydrogel using visible light. Adapted with permission from 117. Copyright 2018, American Chemical Society.

Among the various natural biomaterials, heart-derived dECM (hdECM) is one of the most typic natural bioinks. dECM is obtained from the organ of interest by detergent treatment to remove cells, which preserves the ECM and maintains the architecture of cell-cell interactions (**Figure 6A**). Therefore, hdECM recapitulates most of the chemical cues of native heart tissue to promote cell survival, differentiation, and functionality 107. Jang *et al.* 81 transplanted a cell-free hdECM-based heart patch in the epicardium of a rat MI model to assess the functional benefits of hdECM materials for tissue repair. Less adverse remodeling was observed in the hdECM group than in the blank control group. In contrast, eccentric remodeling of the heart was observed in the control group seven days after implantation. However, the therapeutic concentrations of dECM solution (6-10 mg mL^-1^) had relatively low viscosity, and the bioprinted layers were hard to sustain their previously defined 3D structure. Although previous studies have developed some printing methods to overcome the poor printability of hdECM, there are still two main issues worth attention 74, 81. First, the required concentration of hdECM solution (20 mg mL^-1^) is significantly higher than that used in therapeutic studies, which is limiting because the preparation of hdECM requires extensive harvesting from porcine sources. Second, pure hdECM-printed structures are relatively hard to print and potentially rupture when applied as a patch for heart regeneration due to their low mechanical modulus and fibrous nature 108, 109. Collagen, the major constituent of the ECM, is considered an excellent cell delivery platform for cardiac applications due to its ability to promote cell adhesion and differentiation 110, 111. More importantly, collagen is more readily available to most researchers than dECM. However, collagen also suffers the same printing and mechanical problems as dECM due to its low Young's modulus and viscosity 97. To precisely control the mechanical properties of natural polymers, chemical conjugations of natural biomaterials have been developed 112. For example, methacrylated type-I collagen (MeCol) is obtained by adding methacrylate to the amine-containing side groups of collagen (**Figure 6D**). Collagen provides cell adhesion molecules to ensure the required bioactivity, while methacrylate allows adjustable construct stiffness due to methacrylate photopolymerizability under UV light 97. Similarly, gelatin methacryloyl (GelMA), another widely used material, is gelatin modified with a photopolymerizable methacrylamide group (**Figure 6C**) 107. GelMA is mechanically tunable based on its degree of methacrylation, and its elastic modulus can be adjusted by altering its concentration and printing parameters such as printing temperature, duration of UV light exposure and type, and amount of photoinitiator 113, 114. The choice of photoinitiator is important because every photoinitiator requires a different wavelength of light for crosslinking, and some wavelengths are potentially more damaging to cells. For instance, the suitable wavelengths for 1-[4-(2-hydroxyethoxy)-phenyl]-2-hydroxy-2-methyl-1-propan-1-one (Irgacure 2959) is 365 nm and for lithium phenyl(2,4,6-trimethylbenzoyl) phosphinate (LAP) is 405 nm, respectively. Luckily, a white light system (Eosin Y system) has been developed recently to initiate crosslinking in 5 min without causing any damage to cells 98, 115.

#### Synthetic biomaterials as bioinks

Synthetic polymers have also been widely used as bioinks in 3DBP for CTE applications (**Figure 6E**). In general, synthetic bioinks offer better physical integrity, higher mechanical strength, and enhanced printability compared with natural bioinks. Synthetic polymers potentially have controllable physicochemical properties (e.g., degradation rate, diffusion rate, hydrophobicity) *via* adjustment of their molecular weight or post-printing management 6. He *et al.* 118 fabricated microscale polycaprolactone (PCL) fibers with an average size of 9.5 μm by melt-based electrohydrodynamic (EHD) printing to mimic cardiac collagenous fibers, which guided layer-specific cell orientations. Poly (L-lactic acid) (PLLA) is one of the most extensively studied biodegradable polyesters. PLLA is degraded into water and carbon dioxide by hydrolysis or esterases in the body 119. PLLA-based vascular scaffolds as cardiovascular implants are able to overcome the shortcomings associated with metallic implants, including restricted normal vasomotion and adaptive remodeling of the arterial vessel wall, and bypass surgery 120. However, most synthetic hydrogels are brittle and lack flexibility and elasticity, making it difficult to mimic the softness, stretchability, and elasticity of human soft tissues, such as blood vessels and heart muscles. Xu *et al.* 117 designed a triblock copolymer, PCL-PEG-PCL diacrylate, as the single-component precursor to form a crosslinked hydrogel network (**Figure 6F**). This hydrogel exhibited high flexibility and elasticity, withstanding large deformations from stretching, compression, and twisting without any obvious breakage, and recover quickly from deformation. However, the hydrophobic surface of synthetic polyesters prevents hydration and protein absorption, and the absence of Arg-Gly-Asp (RGD) peptides prevents cell attachment 121, 122. To overcome these limitations, chemical grafting of synthetic polymers has been developed. For example, Costantini *et al.* 123 formulated a tailored bioink with a photocurable semi-synthetic biopolymer (PEG-fibrinogen) that is composed of denatured fibrinogen fragments with covalently attached PEG side chains having a vinyl moiety at their extremities. Shape memory polymers (SMPs) have also been applied to design smart scaffolds. SMPs have the ability to return from a deformed state to their original (permanent) shape when induced by an external stimulus. For example, shape memory polyurethane is widely used to fabricate smart robots or injectable scaffolds because it has controllable structure performance with a tunable shape memory temperature range (-30 to 70 °C) 124. Radisic *et al.* 37 designed an elastic and microfabricated scaffold for functional tissue delivery via injection. Scaffolds and cardiac patches (1 cm × 1 cm) were delivered through an orifice as small as 1 mm and recovered their initial shape following injection without affecting cardiomyocyte viability and function. Xiao *et al*. 125 fabricated a self-adhesive conductive cardiac patch from SMPs to promote electrical signal transduction and improve function in the MI area. Shape memory has shown great potential for invasive delivery via tiny orifices and smart biomedical robots.

#### Hybrid bioinks

**Figure 7:**
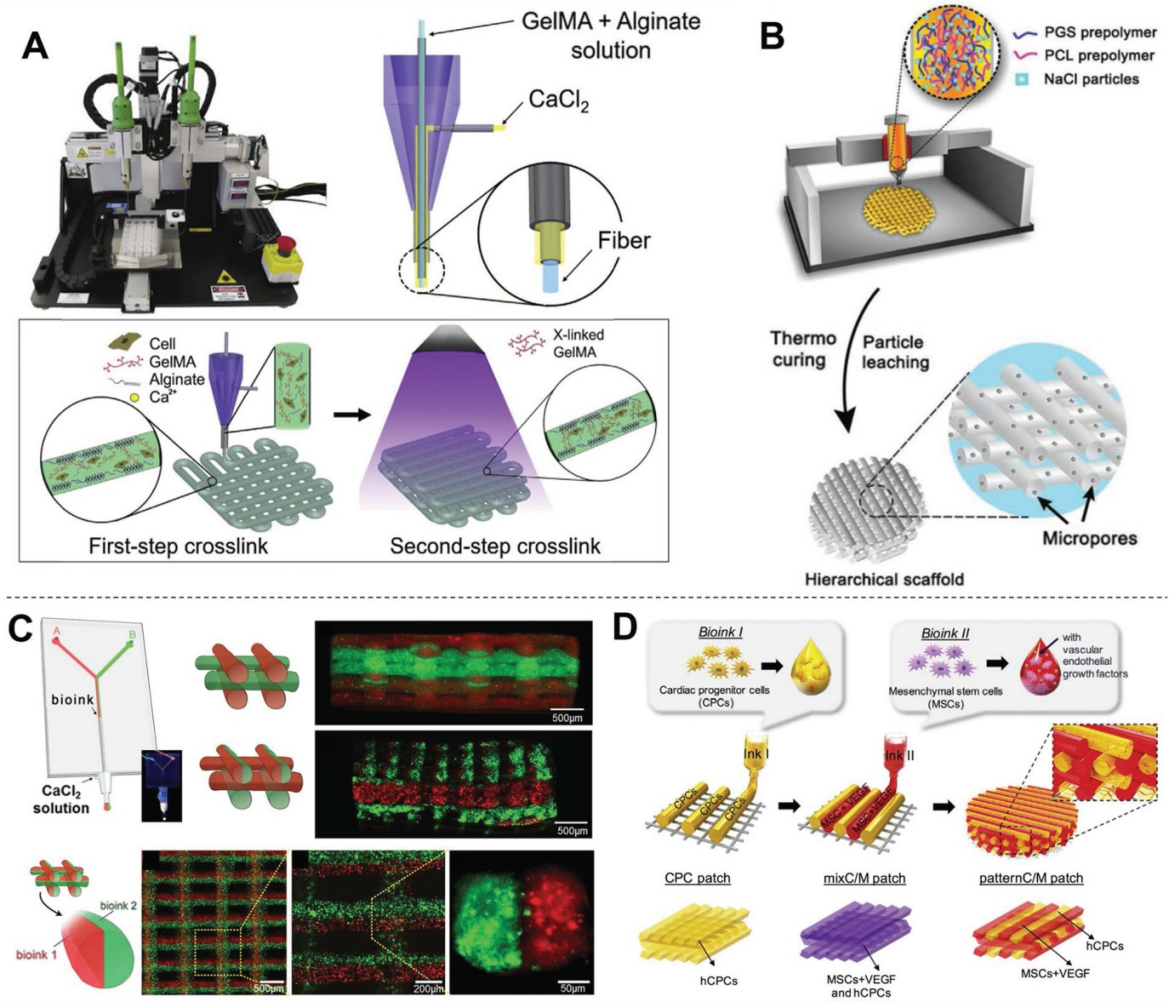
Preparation of hybrid bioinks for 3DBP in CTE. (A) Schematic diagram showing the two-step crosslinking process for alginate-GelMA hybrid bioinks, The alginate component was first physically crosslinked by CaCl_2_ then GelMA was chemically crosslinked *via* UV illumination to stabilize the 3D printed scaffolds. Adapted with permission from 99. Copyright 2016, Elsevier. (B) Schematic diagram illustrating how salt particles can be used as a temporary mechanical support and to facilitate thermoplastic processes, including fused deposition modeling (FDM) 3D printing. Adapted with permission from 135. Copyright 2019, John Wiley & Sons. (C) Schematic diagram of a microfluidic system used to flow two bioinks (containing red and green fluorescent beads) that exited the device through a single extruder. This device opened new routes for the creation of complex and heterogeneous tissue fibers on demand. Adapted with permission from 133. Copyright 2015, John Wiley and Sons. (D) Schematic diagram of a multi-printer system used to fabricate pre-vascularized stem cell patches from multiple cell-laden bioinks and supporting PCL polymer. The printed dual stem cell structure improved cell-to-cell interactions and cell differentiation and promoted functionality for tissue regeneration. Adapted with permission from 81. Copyright 2017, Elsevier.

Hybrid bioinks refer to any permutation of multiple natural or synthetic polymers. Since every biomaterial has merits and faults, hybrid bioinks aim to combine strengths and circumvent weaknesses to form more suitable bioinks for 3DBP. Most hybrid bioinks take advantage of the cell-supportive properties of natural polymers and the mechanical properties and tunability of synthetic polymers. For example, PCL is a widely used biomaterial in 3DBP, but its lack of bioactivity reduces cell affinity and the high hydrophobicity leads to low tissue regeneration rates 126. One solution to overcome these limitations is to add nanoparticles, such as carbon nanotubes (CNTs) 127. Kim *et al.* 94 found that H9c2 cells grown on PCL/CNT composite scaffolds had a higher proliferation rate than those grown on pure PCL scaffolds. This result indicated that the inclusion of CNTs might provide more favorable conditions for the adhesion and proliferation of H9c2 cells. Grafting hydrophilic fragments of synthetic or natural polymers such as acrylates, collagen, and chitosan, to hydrophobic biomaterials has also been explored 128-132. In another study, Bejleri *et al.* 98 combined hdECM with GelMA hydrogel to bioprint cardiac patches for heart repair. The inclusion of hdECM improved the differentiation and reduced the proliferation of neonatal human cardiac progenitor cells (hCPCs) compared with GelMA alone, which in turn may improve the paracrine potential of hCPC-laden GelMA-hdECM patches. Some hybrid bioinks are mixed from multiple biomaterials for optimization of their mechanical properties to for 3DBP. One such strategy involves a second crosslinking reaction to prepare a double-network hydrogel. Alginate crosslinking system is widely used to prepare double-network hydrogels by exploiting the ability of alginate to undergo instantaneous gelation when exposed to Ca^2+^ 123. Moreover, alginates are easily dissolved in the absence of calcium ions. Khademhosseini *et al.* 99 mixed alginate with GelMA (a low-viscosity bioink) to form a hybrid bioink for 3DBP using a coaxial needle (inner: GelMA-alginate bioinks; outer: crosslinker solution) (**Figure 7A**). Instantaneous gelation of the hybrid bioink took place at the tip of the inner needle; therefore, the GelMA-alginate bioink was capable of generating self-supported multi-layered structures. The scaffold was further treated with UV irradiation for photocrosslinking, which improved its structural stability. Colosi *et al.* 133 printed GelMA-alginate following a similar strategy, forming a fully interconnected mesh of deposited fibers that were stacked without signs of vertical collapse. Another strategy is to use nanoparticles as rheology modifier to tune the mechanical properties of the cell-laden fibers to mimic the morphological and mechanical features of native tissue and induce cell spreading. For example, Zhu *et al.* 134 used gold nanorods as a rheology modifier to adjust GelMA bioink. The adjusted gold nanorod-GelMA hydrogel had a Young's modulus of 4.2 ± 0.3 kPa, which was higher than that of pristine GelMA hydrogel (3.75 ± 0.15 kPa) and was adequate for its envisaged application in cardiac tissue implant. You *et al.* 135 adjusted the mechanical properties of poly(glycerol sebacate) (PGS) for stability and ease of extrusion by adding salt particles. The salt was used both as a temporary mechanical support during printing and curing and as a water-soluble porogen for introducing hierarchical micropores. This solution addressed the incompatibility of PGS with typical thermoplastic processes due to its harsh curing conditions of high temperature and high vacuum. In a subsequent study, PGS/PCL/salt composites were printed into cardiac patches with various viscoelastic properties for heart regeneration (**Figure 7B**) 136. Another strategy is to print several bioinks separately using a microfluidic system (**Figure 7C**) or multi-printer devices (**Figure 7D**). The printed synthetic polymers are usually used as support frameworks, which allows mechanically weaker bioinks to be printed on top 81. Meanwhile, Separately printed materials have also been utilized as sacrificial materials to support special hollow structures. For example, Wang *et al.* 137 utilized PCL as the framework, a fibrin-based composite hydrogel as the bioink, and gelatin as the sacrificial material to print a 3D construct in the form of string. Separate dispensing modules were used for each type of hydrogel and PCL during 3D printing., The obtained cardiac tissue constructs showed a spontaneous synchronized beating in culture and a phenomenal response during* in vitro* drug screening studies.

Native myocardium is an electroactive tissue that spontaneously contracts under electric signal propagation 138, 139. Thus, conductivity is an important property for CTE scaffolds. Khademhosseini *et al.* 134 observed that CMs on gold nanorod-incorporated GelMA scaffolds synchronous exhibited beating on day 2, which was much earlier than CMs cultured on pristine GelMA hydrogel (day 5). Currently, various additive conductive materials have been explored to recapitulate the conductivity of native myocardium in CTE. For instance, CNTs are interesting candidate substrates or additives for CTE scaffolds due to their mechanical and electrical properties 140, 141. Izadifar *et al.* 97 bioprinted hybrid cardiac patches from MeCol and alginate. CNT-reinforced hybrid constructs presented significantly higher stiffness and electrical conductivity as well as remarkable growth, proliferation, migration of human coronary artery endothelial cells (HCAECs) over 7 days of culture. In another study, Ho *et al.* 94 mixed CNTs with PCL to print hybrid scaffolds. Incorporation of CNTs reinforced the alignment of the polymer chains, resulting in a gradual enhancement in elastic modulus and hardness as well as slight enhancement in crystallinity, due to interactions with the PCL matrix. PCLCNT nanocomposites with 1%(w/w) CNT showed optimal conductivity for H9c2 cells, leading to a slight increase in their proliferation *in vitro*. In addition, polypyrrole (PPy), a heterocyclic conductive polymer 142, is an excellent candidate additive for CTE scaffolds, showing a host of advantages including stimulus responsiveness, *in vitro* and *in vivo* biocompatibility 143, appropriate chemical stability, large specific surface area, and easy surface modification for incorporation of bioactive molecules 144. Ajdary *et al.* 95 reported drug-loaded printed conductive patches for heart repair based on PGS mixed with PPy and nanofibrillated cellulose. PPy facilitated both cytocompatibility and electrical conductivity (34 ± 2.7 mS cm^-1^) 145 while PGS slowed the degradation of the cardiac patches, making them suitable for long-term drug delivery. In summary, recent advancements in CTE have employed various permutations of multiple natural or synthetic materials to control every property of bioinks in order to mimic native cardiac tissue. These trials have immensely enhanced the variety and possibility of 3DBP for CTE.

### Cell resource

Various types of cells have been used in 3DBP for CTE (**Table 1**) including cell lines, primary myocardial cells, neonatal human cardiac progenitor cells, and stem cell-derived CMs. Established CM cell lines (HL-1, H9c2) are useful alternatives to primary cells for various CTE studies. The H9C2 cell line was originally derived from embryonic rat ventricular tissue 146. H9c2 cells share many properties with primary CMs, including membrane morphology, G protein expression, and electrophysiological properties. However, H9c2 cells are not able to beat 147, 148. In contrast, primary myocardial cells isolated from rat neonatal hearts are widely used to engineer functional cardiac muscle *via* 3DBP technology for regenerative medicine, drug screening, and potentially disease modeling 99. Although primary myocardial cells are excepted to represent the real condition of heart tissue, there is an immunological mismatch between the graft and the host tissue due to the interspecies difference 149, 150. Human neonatal c-KIT-expressing CPCs are harvested from the atrial appendage, which is obtained from pediatric patients aged one week or less undergoing heart surgeries due to congenital heart diseases 98. Agarwal *et al.* 151 showed that progenitor cells could improve the failing right ventricle of neonatal rats subjected to pulmonary banding. However, both primary myocardial cells and hCPCs suffer from shortages in supply. To overcome these challenges, human pluripotent stem cells have been extensively investigated for CTE 152. The stem cells are derived either from developing blastocysts (human embryonic stem cells; hESCs) or from reprogrammed somatic cells (induced pluripotent stem cells; iPSCs) through the addition of transcription factors including Klf-4, Oct 4, Sox 2, and c-Myc 152, 153. These cells could generate unlimited numbers of various types of cells, including functional CMs. Moreover, iPSC-derived CMs have the advantage of overcoming ethical concerns 19. They also allow for the development of personalized medicine with patient-specific implants or drugs because of their ability to proliferate 154, in contrast to the non-proliferating nature of primary CMs. Noor *et al.* 91 utilized iPSC-CMs to bioprint a fully personalized cardiac patch for therapeutic applications. The iPSCs were generated from patient omental stromal cells; therefore, the engineered patches would not provoke an immune response after transplantation, eliminating the need for immunosuppression therapy 88, and have great potential for drug screening in an anatomically appropriate structure. Human pluripotent stem cells are also a predominant source of adult human CMs for clinical therapeutics. However, iPSC-CMs still lack many essential features, such as defined organization and distribution as well as functional transverse tubules 155.

Native heart tissue is composed of multiple types of cells, that each plays a part in CTE. Although CMs are responsible for electrical conduction and generation of contractile force, single CM scaffolds fail to develop tissue constructs when they are cultured alone 156, 157. It has been established that non-muscular heart cells, such as cardiac FBs (CFBs) and vascular ECs, also play a key role in myocardial function. For example, ECs are essential for the vascularization of constructs to match nutrient demands, and cocultures of CMs and ECs in 3D scaffolds were shown to result in functionalized cardiac tissue constructs with increased CM physiology and viability 19. Maiullari *et al.* 96 biofabricated vascularized heart tissue implant with iPSC-CMs and ECs. The bioprinted ECs effectively developed vasculature in the transplanted tissues, which could potentially anastomose with the host vessels. CFBs make up 70% of the cells within the myocardium while only occupying a quarter of the tissue volume, providing essential structural support to CMs and producing most cardiac ECM proteins 158. Many previous studies have formed small self-contracting cardiac muscle strips by combining these two cell populations to mimic the native heart components 159-161. In another study, Arai *et al.* 162 mixed three types of cells to form cell spheroids with high cell densities for 3DBP. Cell spheroids formed from a 50:25:25 mixture of iPSC-CMs/human umbilical vein endothelial cells (HUVECs)/CFBs had a more generalized and homogenous expression pattern than spheroids formed with other cell ratios. By analyzing the time-lapse imaging of cardiac spheroid formation, the authors also demonstrated that the addition of CFBs and HUVECs promoted rapid cell self-organization and enhanced cardiac spheroid stability.

### Applications of 3DBP in CTE

#### 3DBP of heart patches

**Figure 8:**
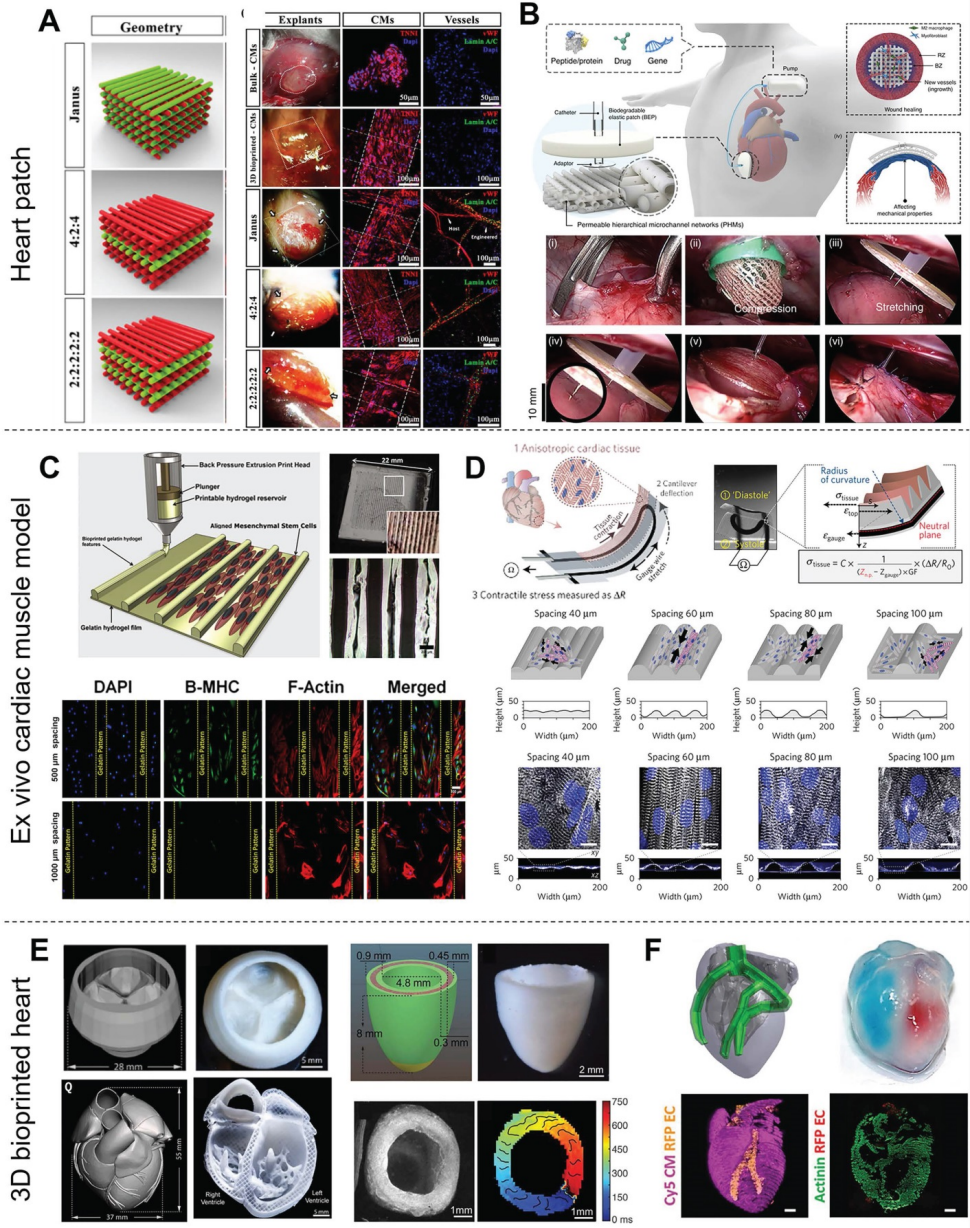
Various applications of 3D-bioprinted constructs for CTE. (A) Left: Schematic illustration of vascularized heart patches printed from iPSC-CM-laden bioink and HUVEC-laden bioink. Right: Representative images of patches after 7 days of culture showing expression of troponin I (red) and connexin 43 (green) in CMs and von Willebrand factor (green) in HUVECs, indicating that a well-developed vascular network was formed in the printed structures. Adapted with permission from 96. Copyright 2018, Springer Nature. (B) Schematic illustration of the perfusable, multifunctional epicardial device (PerMed) consisting of a biodegradable elastic patch, permeable hierarchical microchannel networks and a system to enable delivery of therapeutic agents from a subcutaneously implanted pump (top). The process of PerMed implantation in pigs *via* laparoscopic surgery (bottom). Adapted with permission from 165. Copyright 2021, Springer Nature. (C) Top: Schematic illustration and electron microscopy images of bioprinted microchanneled hydrogels with variable spacing. Bottom: Fluorescence microscopy images assessing the effect of the hydrogels on the alignment, elongation, and differentiation of hMSCs. Adapted with permission from 60. Copyright 2018, IOP Publishing. (D) Design of a cardiac muscle chip with a stress sensor to monitor muscle contraction for applications in drug screening. Adapted with permission from 167. Copyright 2016, Springer Nature. (E) Images of an organ-scale tri-leaflet heart valve (top left), a neonatal-scale human heart (bottom left), and a human cardiac ventricle model (right) printed by FRE, showing the capability of this strategy for precise deposition of bioink. Adapted with permission from 85. Copyright 2019, American Association for the Advancement of Science. (F) Schematic illustration (top left) and photograph (top right) of a heart printed within a support bath and 3D confocal images (bottom) showing its robust structure and perfusable. Adapted with permission from 91. Copyright 2019, John Wiley & Sons.

Left ventricular (LV) remodeling is a pathological process characterized by LV dilation and altered chamber geometry, which is attributable to CM deficiencies. These compensatory mechanisms temporarily ensure adequate cardiac output by increasing stroke volume, at the cost of changing the LV architecture and impairing cardiac contractility. However, CMs have a limited regenerative capacity, and the cardiac ECM is modified and replaced by scar tissue during the progression of heart failure 163. Scar tissue is non-elastic, so it affects heart contraction. Therefore, Heart patches aim to promote functional CMs migration for repairing heart deficiencies instead of scar tissue formation. Bejleri *et al.* 98 combined GelMA and cardiac ECM hydrogel scaffolds with hCPCs to fabricate heart patches with an infill pattern of 90° grids by extrusion printing for myocardial reconstruction. These bioprinted hCPCs had high cell viability and enhanced gene expression of early cardiac transcription factors and the sarcomeric protein troponin T during cell culture. Vessels formed in this patch 14 days after *in vivo* implantation, which indicated that this patch integrated with the native myocardium and allowed for nutrient delivery to the implanted cells. Ajdary *et al.* 95 prepared a 3D-printed curcumin-releasing heart patch from nanofibrillated cellulose, PGS (a representative bioelastomer), and PPy (a conductive polymer). The slow degradation of the cardiac patches was expected to prevent burst release of the drug, making them suitable for long-term drug delivery after MI. Furthermore, many studies have developed heart patches incorporating multiple cells *via* a multiple printer system. For example, Maiullari *et al.* 96 utilized two bioinks containing iPSCs-CMs or HUVECs, respectively, to develop vascularized heart tissue. Ameliorated vascularization was observed for the multi-cell bioprinted structures compared with the controls devoid of endothelial cells. Moreover, the spatial arrangement of the HUVECs played a role in vascularization, and a Janus construct developed a better performing vascular network that was integrated with the host than other printed patterns (**Figure 8A**). Park *et al.* 164 bioprinted a heart patch with human bone marrow-derived mesenchymal stem cells (hBMSCs) and engineered hepatocyte growth factor-expressing MSCs (HGF-eMSCs) for improved vasculogenic potential and enhanced vascular regeneration in MI hearts. The authors further demonstrated that the primed hBMSCs survived longer within a cardiac patch and confirmed cardioprotection, evidenced by substantially higher numbers of viable CMs in the MI hearts. Elasticity is an important parameter of heart patches to match the demands of contractile tissue. You *et al.* 135 used PGS and PCL as a hybrid bioink to print an elastic heart patch for preserving infarcted myocardium. The 3D-printed PGS-PCL patches showed better performance in preserving heart function, reducing infarct size, and increasing heart wall thickness compared with single polymer patches prepared from PCL *or* PGS. In their subsequent study, You's group designed a perfusable, multifunctional epicardial device (PerMed) based on PGS/PCL *via* 3D printing with a subcutaneously implanted drug delivery system and showed the feasibility of minimally invasive surgical PerMed implantation in pigs, which also demonstrated its promise for clinical translation to treat heart disease 165 (**Figure 8B**). The microstructure or pattern also influences the properties of heart patches. Two microarchitectural features defined by the interstrand distance and strand alignment angle have been identified as major parameters for assessing the electrical/mechanical and structural behaviors of 3D‐printed constructs 92. An improper pattern design value usually leads to structurally unstable or lack of transverse conduction 166. In summary, advanced cardiac patches should be considered based on their bioink components and pattern design, which determine regeneration-related properties, such as conductivity, modulus, nontoxicity, and even drug release.

#### 3DBP of *ex vivo* cardiac muscle model

*Ex vivo* cardiac muscle models are one of the primary applications of CTE. They aim to facilitate research on the basic physiology of the heart or to serve as high-throughput drug screening platforms* in vitro*. Organized cell alignment is important for the realization of functional engineered cardiac muscle. Therefore, most CTE studies follow the principle that scaffolds should be designed to induce cardiac cell orientation. In particular, free-form 3DBP technologies allow for easy modification of the constructed pattern given their flexibility compared with other techniques. For example, Tijore *et al.* 60 bioprinted micro-channel patterned gelatin hydrogel scaffolds and then seed neonatal rat CMs on the scaffold for cultivation (**Figure 8C**). Elongated cells with significant *β*MHC expression (a differentiation-related gene) were observed on microchanneled scaffolds with 500 μm spacing. CMs on the microchanneled scaffolds showed more obvious spontaneous beating than those on plain scaffolds. Conversely, on wide microchanneled surfaces (∼800 μm spacing), aligned cells contacting the channel edges tended to have less influence on cells adhered centrally and failed to confer any guidance cues 168, 169. Recently, the concept of “organ-on-a-chip” has trended for its precise control of CTE. Organ-on-a-chip is a microfluidic cell culture device created by microchip manufacturing methods that contains continuously perfused chambers inhabited by living cells arranged to simulate tissue- and organ-level physiology 170. Organ-on-a-chip devices might be more responsive to 3D cell-cell and cell-matrix interactions than *in vitro* 2D cell culture models, meaning they have great potential for drug screening. Moreover, organ-on-a-chip is expected to overcome limitations of traditional animal models such as high costs, time-consuming, and interspecies variation.

Furthermore, the cells in organ-on-a-chip allow for the use of patient-derived iPSCs for tailoring of medical compounds to individual patients 171. However, the need for multiple parameter readouts is a burden on sample analysis. In particular, heart-on-chip requires detection of contraction and monitoring of many chemical combinations. Lind *et al.* 167 printed a sensor-studded cardiac tissue chip to easily monitor heartbeat through the change in electrical resistance. Carbon black nanoparticles were printed into the middle of the chip as an electronic sensor (**Figure 8D**); therefore, stress could be read by calculating the change in resistance, which was much more accessible and straightforward compared with microscopy coupled with optical tracking analysis. Other studies have induced an inverse opal structure to reveal the heartbeat by a change in color 172, 173. These chips were treated with various concentrations of positive or negative inotropic drugs (e.g., isoproterenol) and showed a sensitive response to drug stimulation.

#### 3DBP of engineered hearts

Engineering a whole heart organ with full functions comparable to native tissue is the ultimate goal of CTE to solve the short supply of donated organs. Thanks to the convenience of computer-assisted 3D printing technology, the heart computer-aided design (CAD) models of human hearts were developed from CT and MRI scans or downloaded from the model library. The parameters of the CAD models would be further tuned to make them suitable for 3DBP. However, printing an intact 3D structure with heart geometry is still challenging and is limited by the ability to combine multiple types of cells and fabricate multi-scale structures with different biomaterials. Recently, many elaborate structures have been bioprinted by FRE printing that mimics the macroscopic anatomical heart. For instance, Lee *et al.* 85 printed a tri-leaflet heart valve (28 mm in diameter), a neonatal-scale collagen heart, and a human cardiac ventricle model to demonstrate the precise deposition of their FRESH strategy (**Figure 8E**). The collagen tri-leaflet valve had well-separated leaflets and was robust enough to be handled in air. The authors quantified the flow through the valves and demonstrated <15% regurgitation. Furthermore, HUVECs cultured on unfixed collagen leaflets formed a confluent monolayer. Moreover, the microscale internal structure of the printed neonatal-scale collagen heart, such as trabeculae, matched the architecture defined in the G-code file. In another study, Noor *et al.* 91 printed hearts (height: 20 mm; diameter: 14 mm) from two bioinks containing Cy5-labeled CMs and ECs. The integrity of the different compartments was demonstrated by the injection of blue and red dyes (**Figure 8F**). Moreover, the mechanical properties of the printed hearts closely resembled the properties of decellularized rat hearts. One day after printing, high magnification of the cells comprising the printed heart showed a homogeneous distribution of CMs. In summary, recent developments in whole heart printing present the possibility for 3DBP with high-resolution and precise deposition of boinks. Additionally, the concept of FRE printing offers a theoretical basis on which to build up these complicated crafts, which might make it the most promising strategy for printing advanced tissue scaffolds for a wide range of organ systems 85.

## Perspective and challenges

In the past decade, 3DBP has evolved to become more sophisticated, and some bioprinted human anatomical parts (e.g., ear, nose, hydroxyapatite bone substitutes) have already been used in the clinic 174-176. Despite its significant progress and promise, 3DBP is still incapable of culturing a truly functional heart. One of the challenges is printing resolution. To closely mimic native tissue, bioink should be ideally deposited with a resolution comparable to cell size (5-10 µm). Additionally, to reach clinical applications, thick multi-layered muscle tissue is required. The maximum nutrient/oxygen diffusion distance for cells to survive without vascularization is ~100-200 μm 177. However, it is still challenging to generate controlled vascular tree-like networks. The realization of vascularized CTE might be a barrier for another decade. In recent decades, bioprinting techniques for CTE have significantly developed in structural complexity, but bioprinting of soft materials (e.g., hydrogels) is still immature and many challenges remain 178. Luckily, the emerging concept of using a reversible support bath to enable freeform reversible embedding of suspended hydrogels bioprinting is valid for most low-viscosity materials and makes it possible to print any complicated structure without the design of extra support 85. However, the real heart organ has complex components comprised of multiple cell types, ECM, and multiscale structures for pumping blood, so further development of 3DBP strategies for CTE is needed.

Another research direction in 3DBP is the development of bioinks. Ideal bioinks should be printable, bioactive, biodegradable, stable, affordable, and commercially available with appropriate regulatory guidelines for clinical use 181. However, currently available materials still have deficiencies in these respects. The development of hybrid bioinks is by far the best approach to developing high-quality bioinks. Moreover, smart materials and 4D printing have attached great attention in research communities. In 4D printing, the shape, properties, or functionality of a 3D printed structure evolves overtime when it is exposed to a predetermined stimulus, such as heat 182, 183, humidity 184, 185, light 186, 187, or pH 188. 4D structures can perform specific functions, such as self-folding, drug-releasing, or monitoring, which provide additional functions to scaffolds and generate a massive potential for multiple applications. Breakthroughs in SMPs and new printing control strategies 189 have underlain achievements in 4D printing. However, analysis of the structural mechanics of CAD models before printing is still deficient. In the future, finite element analysis 190, 191 and topology optimization 192, which are widely used in medical equipment design, could be employed to analyze these patches, hearts, and other models to allow for precise design of the material system for structure regulation, and performance optimization.

Another interesting area of research in CTE is the fabrication of 3D structures and functional tissues directly in live animals. For example, Urciuolo *et al.* 193 showed that intravital 3DBP of donor muscle-derived stem cells under the epimysium of hindlimb muscle in mice leads to *de novo* formation of myofibers. Intravital 3DBP takes advantage of commonly available multiphoton microscopes for the accurate positioning and orientation of the bioprinted structures into specific anatomical sites, which has enabled the fabrication of complex structures inside tissues of live mice, including the dermis, skeletal muscle, and brain. Intravital 3DBP might serve as an *in vivo* alternative to conventional bioprinting.

**Figure 9:**
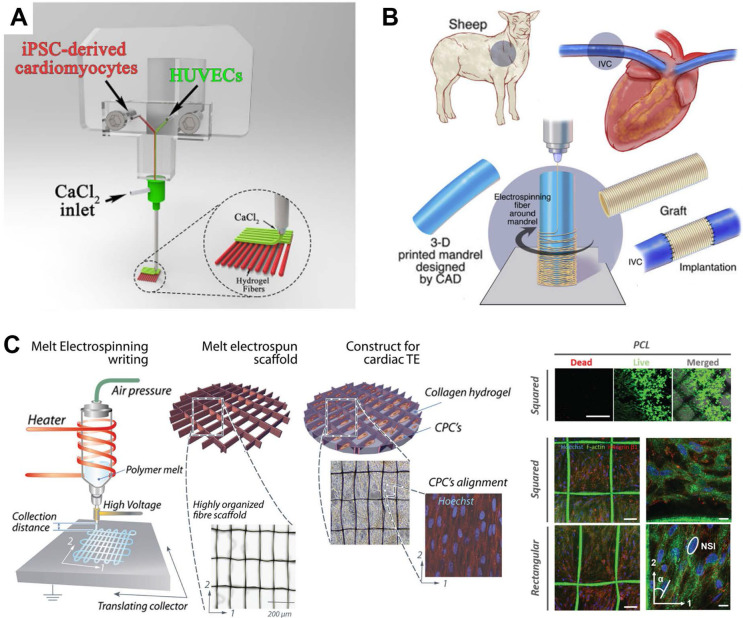
Examples of 3DBP strategies combined with other biofabrication techniques. (A) A microfluidic printing head guaranteeing high-resolution bioprinting generated heterogeneous constructs composed of iPSC-derived CMs and HUVECs with different spatial distributions, enabling fabrication of 3D cardiac tissue models enriched with a vascular network. Adapted with permission from 96. Copyright 2018, Springer Nature. (B) 3DBP was combined with electrospinning to create patient-specific nanofiber tissue-engineered vascular grafts, which were effective in repairing inferior vena cava. Adapted with permission from 179. Copyright 2016, Elsevier. (C) A custom-built melt electrospinning writing device enabled high-resolution (~10 µm) deposition of bioink (left) to print rectangular patterns that promoted CPC alignment (right). Adapted with permission from 180. Copyright 2017, John Wiley & Sons.

Another research trend in CTE is the combination of 3DBP with other biofabrication techniques. Since every printing technique has intrinsic shortcomings, 3DBP has been combined with other biofabrication techniques to benefit from their merit. For example, Maiullari *et al.* 96 combined 3DBP with microfluidic-based printer heads to achieve high-resolution bioprinting of heterogeneous constructs composed of iPSC-derived CMs and HUVECs with different spatial distributions, enabling the fabrication of 3D cardiac tissue models enriched with a vascular network (**Figure 9A**). Fukunishi *et al.* 179 combined 3DBP with electrospinning to create patient-specific nanofiber tissue-engineered vascular grafts (**Figure 9B**). The vascular grafts were implanted as an inferior vena cava interposition conduit in a sheep model. All sheep survived after 6 months without aneurysm formation or ectopic calcification, which indicated the clinical potential of the grafts. Castilho *et al.* 180 reported a melt-based EHD printing technique to deposit fibers with an average diameter of 10 µm to highly defined scaffold structures (**Figure 9C**). The printed cardiac scaffolds had highly organized fiber architectures with a rectangular pattern that promoted CPC alignment and were found to approximate the broad mechanical properties of native myocardial tissue. He* et al.* 118 applied EHD printing techniques to produce micron-scale PCL fibers and sub-micron conductive fibers to mimic the collagenous fibers and conductive Purkinje fibers in native cardiac ECM. CMs on the conductive scaffold showed enhanced synchronous beating compared with those on pure microfibrous scaffolds. Finally, with the rapid developments in bioprinting, common standards for additive manufacturing technologies should be established to normalize 3D-bioprinted products and access more opportunities for clinical trials of therapies.

## Conclusion

3DBP is a promising technique for CTE owing to its ability to print heterogeneous structures and make full use of advanced achievements in cell and material engineering fields. Although there are still many challenges facing CTE for clinical applications, recent developments in printing strategies, such as emerging technologies, new bioinks, and better combinations of cells, will enable 3DBP to satisfy myriads of practical applications, including personalized drug screening, cardiac muscle reconstruction, and organ transplantation.

## Figures and Tables

**Table 1:** Examples of biomaterials fabricated by various 3DBP techniques for cardiac and microvascular tissue engineering applications.

Bioink composition	Bioprinting technique	Cell resource	*In vitro/in vivo* results	Reference
Collagen	FRE Printing	C2C12/ hESC-CMs	High resolution (20 μm) and cell viability (96%). Suspended or hollow structures, such as the neonatal heart, were fabricated directly. Printed scaffolds exhibited micro-porous structures, which were beneficial to vascularization.	Feinberg *et al.* 85,86
Alginate	Extrusion Printing	HCAECs	Interstrand distance and strand alignment angle in the 3D‐printed pattern influenced stiffness, electrical conductivity, and porosity.	Izadifar *et al.*^92^
Gelatin	Extrusion Printing	Neonatal rat CMs/hMSCs	3D-printed microchannels induced hMSC orientation and myocardial lineage commitment, which improved the organization and rhythmic beating of CMs.	Tijore *et al.*^60^
GelMA	DLP Printing	Neonatal rat CMs	Cell shape and orientation in 3D were controlled by engineering scaffold microstructures and encapsulating cells near these geometric cues. Well-aligned myofiber cultured patterns generated 4-10 times the contractile force of less anisotropically patterned constructs.	Liu *et al.*^76^
GelMA	Extrusion Printing	Neonatal rat CMs/CFBs	CM-laden GelMA bioink was significantly more sensitive to extruder pressure than CFB-laden bioinks The ability of CM-laden constructs to form networks was affected by GelMA concentration.	Koti *et al.*^84^
PEGDA	DLP Printing	iPSC-CMs	Microscale continuous optical printing (μCOP) was optimized to achieve miniaturization and promote cardiac tissue maturation. Demonstrated potential for high-throughput *in vitro* drug screening	Ma *et al.*^93^
PCL/CNT	Extrusion Printing	H9c2	Incorporation of CNTs reinforced the alignment of polymer chains, resulting in a slight enhancement in crystallinity, due to interactions with the PCL matrix. PCLCNT nanocomposites with 1%(w/w) CNT showed optimal conductivity and stiffness for the proliferation of H9c2 cells.	Ho *et al.*^94^
PGS/nanocellulose/PPy	Extrusion Printing	H9c2	These cardiac patches fulfilled the requirement of the highly dynamic and functional electroresponsive cardiac tissue given their biocompatibility, biodegradability, mechanical strength, flexibility, and electrical conductivity. The slow degradation of the cardiac patches indicated their suitability for long-term drug release.	Ajdary *et al.*^95^
PCL/hdECM	Extrusion Printing	hCPCs/hMSCs	hdECM might potentiate epicardial-mediated cardiac tissue regeneration followed by migration of Wilms tumor protein 1 positive progenitor cells via epithelial-mesenchymal transition. This 3D pre-vascularized stem cell patch effectively delivered the stem cells *via* the epicardial delivery route.	Jang *et al.*^81^
Alginate/PEG/fibrinogen	Extrusion Printing	iPSC-CMs/HUVECs	Bioprinted endothelial cells effectively developed vasculature in transplanted cardiac tissues, and integrated with the host vasculature.	Maiullari *et al.*^96^
Alginate /MeCol/CNTs	Extrusion Printing	HCAECs	Incorporation of CNTs in MeCol significantly improved the electrical conductivity of the hydrogel and improved cell attachment and elongation. CNTs reinforced hydrogel crosslinking in alginate.	Izadifar *et al.*^97^
GelMA/hdECM	Extrusion Printing	hCPCs/rat-CFBs	Incorporation of hdECM within patches resulted in a 30-fold increase in the cardiogenic gene expression of hCPCs compared to hCPCs grown in pure GelMA patches. Conditioned media from GelMA-hdECM patches show increased angiogenic potential (>2-fold) over pristine GelMA. Patches were retained on rat hearts and showed vascularization over 14 d *in vivo*.	Bejleri *et al.*^98^
GelMA/alginate	Extrusion Printing	Neonatal rat CMs/HUVECs	A microfluidic perfusion bioreactor was designed to complete an endothelialized myocardium-on-a-chip platform for cardiovascular toxicity evaluation.	Zhang *et al.*^99^
GelMA/hdECM	Inkjet Printing	iPSC‐CMs/human-CFBs	The viabilities of cells before and after printing were almost equivalent. High‐density 3D tissues (3.5 × 10^8^ cells cm^-3^) with high cell viability (92.8 ± 1.5%) were produced	Chikae *et al.*^100^

## Abbreviations

hESC-CMs: human embryonic stem cell-derived cardiomyocytes
HCAECs: Human coronary artery endothelial cells
hMSCs: human mesenchymal stem cell
GelMA: gelatin methacryloyl
PEGDA: Poly (ethylene glycol) diacrylate
iPSC-CMs: induced pluripotent cell-derived cardiomyocytes
PCL: polycaprolactone
CNT: carbon nanotube
PGS: poly(glycerol sebacate)
PPy: polypyrrole
hdECM: heart extracellular matrix hydrogel
hCPCs: Neonatal Human Cardiac Progenitor Cell
PEG: Poly (ethylene glycol)
HUVECs: Human Umbilical Vein Endothelial Cells
MeCol: methacrylated collagen
CFBs: cardiac fibroblasts
